# Association of *paired box 6* with high myopia in Japanese

**Published:** 2012-11-17

**Authors:** Masahiro Miyake, Kenji Yamashiro, Hideo Nakanishi, Isao Nakata, Yumiko Akagi-Kurashige, Akitaka Tsujikawa, Muka Moriyama, Kyoko Ohno-Matsui, Manabu Mochizuki, Ryo Yamada, Fumihiko Matsuda, Nagahisa Yoshimura

**Affiliations:** 1 Department of Ophthalmology and Visual Sciences, Kyoto University Graduate School of Medicine, Kyoto, Japan; 2 Center for Genomic Medicine, Kyoto University Graduate School of Medicine, Kyoto, Japan; 3 Department of Ophthalmology and Visual Science, Tokyo Medical and Dental University, Tokyo, Japan

## Abstract

**Purpose:**

The objective of this study was to investigate whether genetic variations in the *paired box 6* (*PAX6*) gene are associated with high myopia in Japanese subjects.

**Methods:**

A total of 1,307 unrelated Japanese patients with high myopia (axial length ≥26 mm in both eyes) and two independent control groups were evaluated (333 cataract patients without high myopia and 923 age-matched healthy Japanese individuals). We genotyped three tag single-nucleotide polymorphisms (SNPs) in *PAX6*: rs2071754, rs644242, and rs3026354. These SNPs provided 100% coverage of all phase II HapMap SNPs within the *PAX6* region (minor allele frequency ≥0.10; r^2^ threshold: 0.90). Chi-square tests for trend and multivariable logistic regression were conducted.

**Results:**

Genotype distributions in the three SNPs were in accordance with the Hardy–Weinberg equilibrium. After adjusting for age and sex, evaluation of cataract control showed a marginal association with high myopia in rs644242 (odds ratio [95% confidence interval]=0.69 [0.49–0.96], p=0.026), and a significant association was observed in healthy Japanese controls (0.79 [0.66–0.96], p=0.015). We pooled two control cohorts to evaluate the association. This analysis revealed a strong association between rs644242 and high myopia (0.78 [0.65–0.92], p=0.0045). The rs644242 A allele was a protective allele for development of high myopia. Subanalysis also revealed that rs644242 was significantly associated with extreme high myopia (0.78 [0.64–0.95], p=0.0165). The other two SNPs did not show a significant association with this condition.

**Conclusions:**

The current study showed a significant association of *PAX6* with high and extreme myopia in Japanese participants. The A allele of rs644242 is a protective allele.

## Introduction

Myopia is the most common visual disorder in the world and presents major public health concerns, especially in East Asian populations. Eyes with long axial lengths (≥26 mm) or a high degree of myopic refractive error (≤−6 diopter [D]) were diagnosed with high myopia [1]. High myopia is associated with various ocular complications [2], and pathological myopia is one of the leading causes of legal blindness in developed countries [3-5]. Therefore, clarifying the pathological pathway that leads to high myopia and developing methods for preventing or delaying its onset are important.

Myopia is a complex disease caused by environmental and genetic factors. Although linkage analysis studies have revealed more than 20 myopia-susceptibility loci and various candidate genes have been evaluated, most of these genes were not consistently responsible for high myopia. Recently, several groups performed genome-wide association studies (GWAS); we determined a susceptible locus at 11q14.1 [6] and 5p15 [7], while studies of Caucasians revealed myopia-susceptibility loci on chromosome 15 [8,9]. We demonstrated the association of these susceptibility loci on chromosome 15 with high myopia in Japanese [10], and a Chinese study successfully replicated the association between high myopia and the *catenin δ2* (*CTNND2*) gene polymorphism in the susceptibility loci 5p15 we determined [11]. However, although the C allele of *CTNND2* single nucleotide polymorphism (SNP) rs6885224 was a risk allele for high myopia in our study, the replication study showed this allele was protective against high myopia. Since the expression of the catenin δ2 protein is regulated by transcription factor Pax6 [12] and *PAX6* is another myopia-susceptibility gene, *PAX6* and *CTNND2* might cooperatively affect myopia development. Although several studies have examined the association between *PAX6* and myopia, whether *PAX6* is a susceptibility gene for myopia remains controversial [13-20]. To determine whether *PAX6* is associated with high myopia, we conducted a large-cohort case–control study of Japanese participants.

## Methods

All procedures adhered to the tenets of the Declaration of Helsinki. The Institutional Review Board and the Ethics Committee of each participating institute approved the protocols. All the patients were fully informed of the purpose and procedures of the study, and written consent was obtained from each patient.

### Patients and control subjects

In total, 1,307 unrelated Japanese patients with high myopia from the Kyoto University Hospital, Tokyo Medical and Dental University Hospital, Fukushima Medical University Hospital, Kobe City Medical Center General Hospital, and Ozaki Eye Hospital were included in the study. Comprehensive ophthalmic examinations were conducted on all the patients, which included dilated indirect and contact lens slit-lamp biomicroscopies, automatic objective refractions, and measurements of axial length using applanation A-scan ultrasonography or partial coherence interferometry (IOLMaster; Carl Zeiss Meditec, Dublin, CA). An axial length of at least 26 mm in both eyes confirmed the patient had high myopia.

Two control cohorts were recruited for this study. The first cohort was categorized as the selected control group, and comprised 333 cataract patients with axial lengths of less than 25.0 mm in both eyes (control 1). These patients were recruited from the Department of Ophthalmology at Kyoto University Hospital, the Ozaki Eye Hospital, the Japanese Red Cross Otsu Hospital, and the Nagahama City Hospital. In this group, the mean age ± standard deviation (SD) was 75.2±7.9 years; 37.2% were men, and 59.5% were women. Axial length was measured with applanation A-scan ultrasonography or partial coherence interferometry before cataract surgery, and post-surgery, a dilated fundus examination was performed. If the fundus examination revealed that myopic changes had occurred, such as lacquer cracks/peripapillary atrophy, staphyloma, or choroidal neovascularization, the subject was eliminated from the group.

The second cohort was recruited as a general-population control. In total, 923 healthy unrelated Japanese individuals were recruited from the Aichi Cancer Center Research Institute (control 2). Only individuals at least 35 years of age were selected to participate in this group, meaning that the controls were age-matched with the high-myopia cohort. The mean age ± SD of this cohort was 56.9±11.4 years (p=0.855 compared with the high-myopia cohort); 39.3% were men, and 60.7% were women.

### Genotyping and statistical analyses

Genomic DNAs were prepared from peripheral blood using a DNA extraction kit (QuickGene-610L; Fujifilm, Minato, Tokyo, Japan) according to the manufacturer’s protocol, and the A260/A280 optical density was measured. Extracted DNA was stored at -80 °C until used. Three tag SNPs (rs2071754, rs644242, and rs3026354) were selected using Tagger software, and provided 100% coverage for all common phase II HapMap SNPs (minor allele frequency: >10%; Build: 36.1) within a 22.4-kb region that covered the *PAX6* gene on chromosome 11 (r^2^ threshold: 0.90). The samples from patients with high myopia and the cataract controls were genotyped using a commercially available assay (TaqMan SNP assay with the ABI PRISM 7700 system; Applied Biosystems, Foster City, CA). The individuals recruited from the Aichi Cancer Center Research Institute were genotyped using Illumina HumanHap 610 Chips (Illumina Inc., San Diego, CA). The genotype for rs3026354 was obtained from imputed data using MACH software because it was not included in the Illumina BeadChip. Phase II HapMap (Build: 36.1) was referred to for reference sequences.

Deviations from the Hardy–Weinberg equilibrium (HWE) in genotype distributions were assessed for each group using the HWE exact test. The chi-square test for trend or its exact counterpart was used to compare the genotype distributions of the two groups. Multiple regression and logistic regression analysis were performed to adjust for age and sex. These statistical analyses were conducted using Software R (R Foundation for Statistical Computing, Vienna, Austria). A p value of less than or equal to 0.05 was considered statistically significant. Bonferroni correction was used for multiple comparisons.

## Results

The demographics of the study population are shown in Table 1. The mean axial length of the 2,614 eyes with high myopia was 29.17±1.84 mm. Of the eyes in this group, 1,878 (71.8%) were phakic, with a mean refraction of −12.71±4.57 D. In the control 1 group, the mean axial length of the 666 eyes was 22.87±0.80 mm, and the mean refraction of the phakic eyes in this group was −0.355±2.96 D.

**Table 1:** Characteristics of the study population.

Population characteristics	High myopia*	Case		Control	
		Control 1†	P value	Control 2	P value
Patients (n)	1307	333		923	
Age (mean±SD; years)	57.1±15.0	75.2±7.9	<0.0001‡	56.9±11.4	0.8549‡
Sex (n)					
Male	427 (32.7%)	124 (37.2%)	0.05626§	363 (39.3%)	0.0015§
Female	879 (67.3%)	198 (59.5%)		560 (60.7%)	
Axial length (mm±SD)					
Right eyes	29.23±1.85	22.84±0.81	NA		
Left eyes	29.10±1.82	22.88±0.78	NA		
Refraction of the phakic eyes (D)					
Right eyes	−12.86±4.44	−0.411±3.15	NA		
Left eyes	−12.57±4.71	−0.296±2.77	NA		

* Axial length >26.00 mm in both eyes. † Individuals who underwent cataract surgery and who had an axial length of <25.00 mm in both eyes.‡ Unpaired t test. Compared with the high-myopia group. § Chi-square test. Compared with the high-myopia group. SD: standard deviation, D: diopter, NA: Not applicable.

The genotype counts, associations, and odds ratios (ORs) for the three SNPs in the high-myopia and control groups are shown in Table 2. The genotype distributions of the three SNPs were in HWE (p>0.05). After corrections for age and sex differences had been made, based on a logistic regression model, rs644242 showed a marginal association (p=0.026) with high myopia when evaluated with control 1 (n=333), and a significant association (p=0.015) when evaluated with control 2 (n=923); further analysis demonstrated that this association was still significant after Bonferroni correction. For the high-myopia group, the odds ratios were 0.69 (95% confidence interval [CI]: 0.49–0.96) for the rs644242 A allele when evaluated with control 1, and 0.79 (95% CI: 0.66–0.96) when evaluated with control 2. Chi-square tests for the trend also showed that rs644242 was significantly associated with high myopia when this group was evaluated with control 2 (p=0.015). The two other SNPs did not have any significant associations with the condition.

**Table 2:** Genotype counts, associations, and odds ratios in participants with high myopia and control participants.

Single nucleotide polymorphisms	Genotype	High myopia	Control 1				Control 2			
		n	n	Nominal p value*	Adjusted p value†	Adjusted OR (95% CI)	n	Nominal p value*	Adjusted P value†	Adjusted OR (95% CI)
rs2071754 (C/T)	CC	326	90	0.61	0.26	1.12 (0.92-1.38)	232	0.485	0.497	1.04 (0.93-1.17)
	CT	632	156				466			
	TT	344	87				225			
rs644242 (C/A)	CC	1052	258	0.12	0.026	0.69 (0.49-0.96)	710	0.0153	0.0152	0.79 (0.66-0.96)
	CA	237	68				195			
	AA	14	7				18			
rs3026354 (A/G)	AA	544	142	0.33	0.78	1.03 (0.83-1.29)	376	0.611	0.638	0.97 (0.86-1.10)
	AG	590	155				421			
	GG	171	34				126			

* Differences in the observed genotypic distribution were examined by a chi-square test for trend. † Age and sex adjustment were performed based on a logistic regression model. CI: Confidence interval, OR: Odds ratio.

Since the allele frequency and the genotype frequency of the three SNPs were not significantly different (p>0.20) between control 1 and control 2, we pooled the controls for further analysis (Table 3). The genotype distributions in the pooled control were still within HWE. This analysis revealed that the rs644242 polymorphism was strongly associated with high myopia. The p value of a chi-square test for the trend was 0.011, and was 0.0045 after adjusting for age and sex with a logistic regression model. Since previous studies have reported on SNP associations with extreme myopia, the genotype distributions of the three SNPs between the extreme myopia cases were compared (axial length ≥28 mm in both eyes) as a pooled control. After age and sex adjustment and Bonferroni correction, this analysis also showed a significant association between rs644242 and extreme myopia (p=0.0165). The OR of this analysis was similar to the OR for the high-myopia analysis (0.78 [95% CI:0.64–0.95]). To investigate whether there are more appropriate genetic association models, we applied other possible ones: dominant, recessive, and codominant. However, we did not find a more significant association than the additive model.

**Table 3:** Genotype counts, associations, and odds ratios in patients with high myopia, extreme myopia and pooled control participants.

SNP	Genotype	Pooled control				High myopia				Extreme myopia				
		n	Genotype frequency p value*	Allele frequency p value*	HWE P value	n	Nominal p value†	Adjusted p value‡	Adjusted odds ratio (95% confidence interval)	n	Nominal p value†		Adjusted p value‡	Adjusted odds ratio (95% confidence interval)
rs2071754	CC	322	0.53	0.99	0.74	326	0.44	0.343	1.06	196	0.327		0.317	1.07
(C/T)	CT	622				632			(0.94–1.18)	397				(0.94–1.21)
	TT	312				344				215				
rs644242	CC	968	0.95	0.95	0.15	1052	0.0105	0.00445	0.78	651	0.0294		0.0165	0.78
(C/A)	CA	263				237			(0.65–0.92)	149				(0.64–0.95)
	AA	25				14				8				
rs3026354	AA	518	0.28	0.22	1	544	0.99	0.834	0.99	346	0.656		0.585	0.96
(A/G)	AG	576				590			(0.88–1.11)	359				(0.84–1.10)
	GG	160				171				104				


* The difference in genotype and allele frequency between control 1 and control 2 were analyzed using a chi-square test. † Differences in the observed genotypic distribution were examined by chi-square test for trend. ‡ Age and sex adjustment were performed based on a logistic regression model. SNP: Single-nucleotide polymorphism, HWE: Hardy–Weinberg equilibrium

Comparisons between the results of the current study and those of previous studies are summarized in Table 4. The current study is the first study to prove significant associations between a *PAX6* SNP and high myopia and extreme myopia.

**Table 4:** Summary of previous reports that evaluated an association between *PAX6* and high myopia.

Author (year)	Definition of cases		Cases	Controls	Reported single-nucleotide polymorphisms*			Remarks
	Criteria of high myopia	Affected eye			rs667773‡ rs644242 rs662702	rs3026390 rs3026393 rs2071754	rs3026354 rs628224	
Tsai et al., 2008	SE<6 D	Both	255	87	n.s.	-	-	Significant association of AC- and AG-repeat lengths in the P1 promoter
Ng et al., 2009	SE≤6 D	-	379	349	n.s.	-	-	
Han et al., 2009	SE<6 D	Both	FBAT† with 164 nuclear family	n.s.	p=0.0011	n.s.		Significant association in haplotype analysis
Liang et al., 2011	SE≤6 D	At least one eye	1083	1096	n.s.	n.s.	n.s.	
Jiang et al., 2011	SE≤8 D	Both	300	300	n.s.	n.s.	n.s.	
	SE≤8 D	Both	299	299	-	n.s.	-	
Current Study	AL≥26 mm	Both	1307	1256	p=0.0045	n.s.	n.s.	
Tsai et al., 2008	SE<10 D	Both	67	87	p<0.001	-	-	
Liang et al., 2011	SEM≤11 D	At least one eye	55	619	p=0.0074	n.s.	n.s.	
Current Study	AL≥28 mm	Both	810	1256	p=0.0165	n.s.	n.s.	

* Single-nucleotide polymorphism pairs with r2 ≧ 0.90 in HapMap Phase II are in the same column. † Family-based association test ‡ Although this single-nucleotide polymorphism is not included in HapMap SNP, it is reported to be in strong linkage disequilibrium (r2=0.92) with rs644242. D: diopter, n.s.: Not significant, SE: Spherical equivalent, AL: Axial length.

## Discussion

In the present study, using a relatively large cohort of 2,563 individuals, we showed that *PAX6* is associated with high and extreme myopia in Japanese. The minor A allele of rs644242 was a protective allele for high and extreme myopia.

The association of *PAX6* with common myopia was first evaluated in a Caucasian cohort. Although genome-wide linkage scans in a twins study suggested the *PAX6* region was strongly linked to common myopia, further case–control studies using tag SNPs rejected the hypothesis of an association between *PAX6* and common myopia [13-15]. Regarding high myopia, although Han et al., in a Chinese nuclear family study, reported that two SNPs in *PAX6* were associated with the condition [17], the subsequent case–control study did not replicate these associations, while haplotype analyses using 16 SNPs revealed the association [19]. Two Chinese reports also denied an association of *PAX6* with high myopia, while the subgroup analysis showed *PAX6* was associated with extreme myopia [16,20]. However, only 67 and 55 cases were used in these subgroup analyses, respectively, and therefore, caution should be applied when interpreting the findings, as pointed out by Zayats et al. [21].

Table 4 summarizes the SNPs that have been evaluated previously to discover if *PAX6* is associated with high/extreme myopia. Rs667773 and rs662702 are reportedly in strong linkage disequilibrium with rs644242 [20], which showed significant association with high/extreme myopia in the present study. The association of these SNPs with extreme myopia was reported by Tsai et al. and Liang et al. [16,20], as well as in the current study, and the direction was the same in these three studies.

There are three possible reasons previous studies did not identify the association of rs644242 (or SNPs in strong linkage disequilibrium with rs644242) with high myopia. First, the parameter used to define high myopia was axial length, while all of the previous studies used standard error of the mean (SEM). Currently, *PAX6* is considered the “master gene” in eye development, owing to the gene’s pivotal role during the induction of lens and retina differentiation [22]. At an early stage of eye development, *PAX6* expression alone forms the eyeball and, with *SOX2*, affects the crystalline lens [23]. Hence, using SEM to define high myopia, which is affected by lens and eye shape, does not convey the direct effects of *PAX6*. However, high myopia defined by axial length, which is determined by changes to the shape of the eye only, demonstrates the direct effects of *PAX6*. This is why previous studies showed a significant association only in extreme myopia; almost all cases of extreme myopia present an abnormal eye shape. The second reason for the discrepancy between the studies is the number of cases. All the previous studies (except the study by Liang et al. [20]) had fewer than 600 participants [16,17,19], which is less than half the number of cases we included in our study. The variance in the inclusion criteria for patients with high myopia is the last possible reason previous studies failed to identify the association. Although Liang et al. included more than 1,000 cases, the researchers defined high myopia as SEM no greater than −6 D in at least one eye [20]. Considering the effect of the *PAX6* gene, the current inclusion criterion, which is to enroll patients who have two highly myopic eyes, is more suitable for selecting genetic-dependent high myopia. Indeed, the inclusion criteria of other studies are the same as in the current study [16,17,19].

**Figure 1:**
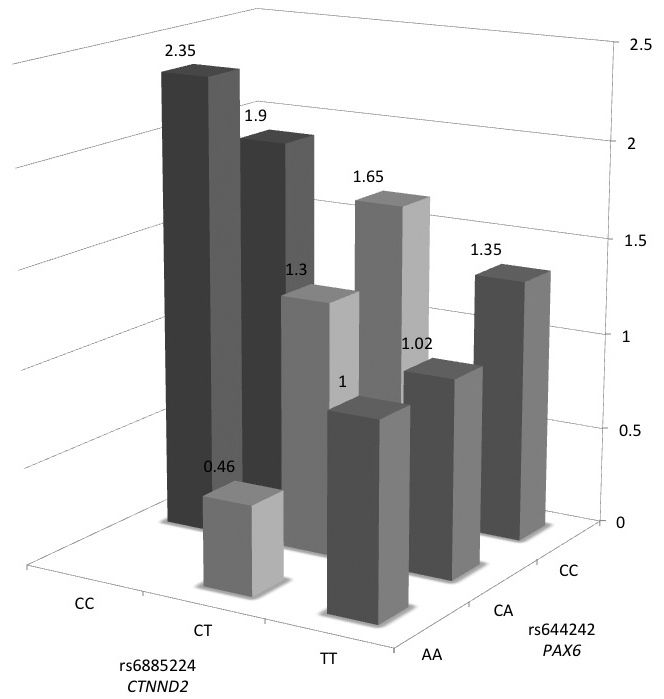
Collaborative effect of *CTNND2* rs6885224 and *PAX6* rs644242 on high myopia. The odds ratio of each genotype-pairs was calculated adjusting for age and sex. Patients with both the rs644242 AA genotype (non-risk homo) and the rs6885224 TT genotype (non-risk homo) are set as the reference (odds ratio=1.0). The number of subjects with rs6885224 CC and rs644242 CC were 55 in the case group and 42 in the control group, 286 in the case group and 252 in the control group with rs6885224 CT and rs644242 CC, 392 in the case group and 416 in the control group with rs6885224 TT and rs644242 CC, 15 in the case group and nine in the control group with rs6885224 CC and rs644242 CA, 70 in the case group and 78 in the control group with rs6885224 CT and rs644242 CA, 76 in the case group and 108 in the control group with rs6885224 TT and rs644242 CA, three in the case group and nine in the control group with rs6885224 CT and rs644242 AA, and six in the case group and nine in the control group with rs6885224 TT and rs644242 AA.

Recently, our GWAS showed that *CTNND2* is a susceptibility gene for high myopia [7]. *CTNND2* encodes catenin δ2, also known as δ-catenin. Catenin δ2/δ-catenin belongs to the catenin δ1/p120-catenin protein family, which regulates cell adhesion and intracellular signaling pathways [24-26]. P120-catenin and β-catenin bind to the cytoplasmic tail of cadherin, which stabilizes the adherence junctions composed of cadherin, p120-catenin, β-catenin, α-catenin, and the actin cytoskeleton. δ-catenin competes with p120-catenin for interaction with cadherin and destabilizes the adherens junction [26,27]. In addition, δ-catenin can also affect the gene expression of other molecules associated with the wingless (Wnt)/β-catenin signaling pathway [28]. Since *CTNND2* expression is regulated by Pax6 [12], and that the distribution of Pax6 and δ-catenin/catenin δ2 is remarkably similar [29,30], the collaboration of *PAX6* and *CTNND2* might be associated with myopia. In genetic studies on age-related macular degeneration (AMD), its association with the *CFH* gene led to the discovery that other molecules in the complement pathway were also associated with the condition, such as *C2/CFB*, *C3*, and *CFI* [31-34]. Similar to these collaborative associations of several complement factors to AMD, molecules associated with the adherence junction and Wnt/β-catenin signaling might contribute to the development of myopia. When we calculate the odds ratio of each genotype-pairs of *PAX6* and *CTNND2* using samples shared between the present study and our previous study [7], the C allele of *CTNND2* rs6885224 seems to be a risk allele for high myopia in populations with the CC/CA genotype in *PAX6* rs644242, while the T allele of CTNND2 rs6885224 seems to be a risk allele in populations with the AA genotype in *PAX6* rs644242 (Figure 1). However, since the number of patients with the *PAX6* AA genotype are small, replication studies are needed.

In conclusion, we proved the significant association of rs644242 in *PAX6* with high and extreme myopia. The A allele for rs644242 is protective for high and extreme myopia, and the collaboration of *PAX6* and *CTNND2* might be associated with the development of this condition. The adherens junction and Wnt/β-catenin signaling are possible attractive targets for further study of myopia development.
